# Identification and Validation of a PEX5-Dependent Signature for Prognostic Prediction in Glioma

**DOI:** 10.3390/biom14030314

**Published:** 2024-03-06

**Authors:** Xuhui Qin, Bing Wang, Xia Lu, Yanyang Song, Wei Wang

**Affiliations:** Department of Human Anatomy, School of Basic Medicine, Tongji Medical College, Huazhong University of Science and Technology, Wuhan 430070, China

**Keywords:** glioma, peroxisome, PEX5, prognosis, immune microenvironment

## Abstract

Gliomas, the most prevalent and lethal form of brain cancer, are known to exhibit metabolic alterations that facilitate tumor growth, invasion, and resistance to therapies. Peroxisomes, essential organelles responsible for fatty acid oxidation and reactive oxygen species (ROS) homeostasis, rely on the receptor PEX5 for the import of metabolic enzymes into their matrix. However, the prognostic significance of peroxisomal enzymes for glioma patients remains unclear. In this study, we elucidate that PEX5 is indispensable for the cell growth, migration, and invasion of glioma cells. We establish a robust prognosis model based on the expression of peroxisomal enzymes, whose localization relies on PEX5. This PEX5-dependent signature not only serves as a robust prognosis model capable of accurately predicting outcomes for glioma patients, but also effectively distinguishes several clinicopathological features, including the grade, isocitrate dehydrogenase (*IDH*) mutation, and 1p19q codeletion status. Furthermore, we developed a nomogram that integrates the prognostic model with other clinicopathological factors, demonstrating highly accurate performance in estimating patient survival. Patients classified into the high-risk group based on our prognostic model exhibited an immunosuppressive microenvironment. Finally, our validation reveals that the elevated expression of *GSTK1*, an antioxidant enzyme within the signature, promotes the cell growth and migration of glioma cells, with this effect dependent on the peroxisomal targeting signal recognized by PEX5. These findings identify the PEX5-dependent signature as a promising prognostic tool for gliomas.

## 1. Introduction

Gliomas are the most common and deadly type of cancer in the central nervous system in adults, accounting for 80% of all malignant brain tumors [1]. Glioblastoma (GBM), an extremely aggressive form of glioma, makes up 50% of newly diagnosed glioma cases and has a median overall survival (OS) of less than two years [2]. Despite advances in standard treatments like surgery, radiotherapy, and chemotherapy, therapeutic resistance remains a significant challenge due to the highly infiltrative nature of malignancy [3]. To address this, previous studies have identified various molecular markers, such as mutations in the isocitrate dehydrogenase 1 and 2 genes (*IDH1* and *IDH2*, collectively referred to as *IDH*) and the co-deletion of 1p/19q, to aid in molecular pathological diagnosis, treatment options, and prognostic assessment [4]. However, the prognosis for glioma patients remains poor, necessitating the development of an improved prognostic model for the disease.

Peroxisomes are crucial single membrane-bound organelles present in all eukaryotic cells. Their importance for mammalian physiology is underscored by the existence of severe inherited human diseases resulting from the complete or partial loss of peroxisomal functions [5]. Peroxisomes are involved in essential cellular metabolism, most notably FA oxidation as well as the synthesis and breakdown of ROS [5,6]. Additionally, peroxisomes participate in various other processes such as glyoxylate detoxification, the synthesis and breakdown of ether phospholipids, and amino acid metabolism [5,6].

To enable these metabolic pathways, peroxisomes contain a variety of metabolic enzymes in the matrix. The import of matrix proteins is typically facilitated by peroxisomal targeting signals (PTSs), such as PTS1, which comprises a non-cleaved, C-terminal tripeptide, SKL, or its conserved variants [7], or PTS2, which consists of a nonapeptide sequence, (R/K)(L/V/I/Q)XX(L/V/I/H/Q)(L/S/G/A/K)X(H/Q)(L/A/F), located near the N terminus of the cargo protein [8]. PEX5 functions as the receptor for the recognition and import of PTS1-containing proteins [9,10], while PEX7 is the receptor for PTS2 proteins. Remarkably, PEX5 acts as a co-receptor for the import of PTS2 proteins by binding to the PEX7-cargo complexes [11,12]. Additionally, membrane proteins harbor targeting signals (mPTS), recognized by the receptor PEX19 [13].

One of the hallmarks of glioma progression is metabolic reprogramming, which allows tumor cells to adapt and survive in a hostile microenvironment [14]. In gliomas, alterations in metabolic pathways, such as fatty acid, lipid, and redox metabolism, are not only a consequence of genetic and epigenetic changes, but they also contribute to tumor growth, invasion, and therapy resistance [14,15]. Accumulating evidence indicates that peroxisomes actively participate in cancer, a disease characterized by abnormal metabolism [16]. A significant proportion of gliomas carry mutations in the *IDH*1 and *IDH*2 genes [17]. It is worth mentioning that *IDH*1 is partially localized within peroxisomes [18]. Several studies have linked the expression of peroxisomal genes and proteins to the malignancy grade of gliomas [19,20,21]. However, the prognostic significance of peroxisomes for glioma patients remains unclear.

In our study, we focused on the receptor PEX5, as it plays a crucial role in the matrix import of major peroxisomal enzymes. We developed a PEX5-dependent prognosis model and evaluated its sensitivity, specificity, and accuracy in predicting the survival of glioma patients. Furthermore, we knocked out the *PEX5* gene and assessed the essentiality of *PEX5* in glioma cell growth, migration, and invasion.

## 2. Materials and Methods

### 2.1. Data Acquisition and Processing

We retrieved expression data and clinical information for glioma patients from multiple public databases. Specifically, we obtained the TCGA RNA-seq datasets from the UCSC Xena website, which consisted of 529 lower-grade glioma (LGG) samples and 173 glioblastoma (GBM) samples. Additionally, we downloaded two RNA-seq datasets, CGGA_693 (693 samples) and CGGA_325 (325 samples), from the Chinese Glioma Genome Atlas. We also acquired the GSE16011 microarray dataset (284 samples) from the Gene Expression Omnibus (GEO) database.

For the RNA-seq cohorts, we obtained both the FPKM (fragments per kilobase of exon model per million mapped fragments) values and raw count data for expression analysis. To ensure the quality of the prognosis model analysis, we filtered out patient data with missing survival time or status information. After applying this criterion, we obtained a total of 691, 657, 313, and 131 pieces of glioma patient data for the TCGA, CGGA_693, CGGA_325, and GSE16011 cohorts, respectively. The detailed clinicopathological characteristics of glioma patients are summarized in Tables S1–S4.

### 2.2. Construction of the PEX5-Based Prognostic Gene Signature

Identification of PEX5-dependent peroxisomal metabolic enzymes was carried out by searching in the Peroxisome database (http://www.peroxisomedb.org/home.jsp, (accessed on 22 February 2024)) and selecting those with PTS1 or PTS2 signals. The resulting genes were then subjected to least absolute shrinkage and selection operator (LASSO) regression analysis using the glmnet R package. Multivariate Cox regression analysis was subsequently performed with the survival R package to establish an optimal PEX5-based risk signature. A prognostic risk score was generated for each patient with the following formula: risk score = expression level of gene1 × j1 + expression level of gene2 × j2 + … + expression level of genex × jx, where j represents the coefficient. We used TCGA data as the training cohort, while CGGA_693, CGGA_325, and GSE16011 data were used as validation cohorts.

### 2.3. Survival Analysis

The glioma patients were categorized into low- and high-risk groups based on their median risk score. A risk plot was generated using an in-house “risk_plot” R function to illustrate the distribution of survival status of the patients and the expression of the risk signature genes in the different risk groups. To evaluate the sensitivity and specificity of the risk signatures in predicting outcomes, we established time-dependent receiver operating characteristic (ROC) curves using the R package “survivalROC”. For the ROC analysis, a higher area under the curve (AUC) indicates better accuracy. The AUC values range from 0.5 to 1.0, where 0.5 represents random chance, and 1.0 represents perfect discrimination. Generally, an AUC value greater than 0.7 is considered a reasonable estimate [22]. To compare the OS between different groups of glioma patients, we conducted Kaplan–Meier survival analyses using the R “survival” and “survminer” packages.

### 2.4. Independent Prognostic Role of the Risk Signature

To determine whether the PEX5-based risk score and other clinicopathological factors were independent prognostic factors for glioma patients, univariate and multivariate Cox regression analyses were conducted using the “survival” R package. These clinicopathological factors included age, gender, tumor grade, *IDH* mutation status, 1p19q codeletion status, and MGMT promoter methylation status. Results were presented using forest plots generated by the “forestplot” R package.

### 2.5. Construction of the Nomogram

To develop a prognostic nomogram for glioma patients, the “rms” R package was utilized. The nomogram included the PEX5-based risk score and the aforementioned clinicopathological factors, and was used to predict survival probabilities at 1, 3, and 5 years. The accuracy of the nomogram was evaluated through calibration curves and decision curve analysis (DCA) for the one-, three-, and five-year survival rates of glioma patients. DCA is a method for evaluating the clinical benefit of alternative models [23]. In the context of nomograms, DCA evaluates net benefits at various threshold probabilities. To provide a meaningful comparison, two reference curves are typically plotted: the treat-all-patients scheme, representing the highest clinical costs, and the treat-none scheme, indicating no clinical benefit.

### 2.6. Differential Expression Gene (DEG) and Functional Enrichment Analysis

Raw count data from the three RNA-seq cohorts (TCGA, CGGA_693, and CGGA_325) was obtained, and genes with expression detected in at least half of the samples were selected for DEG analysis using the “limma” R package between the low- and high-risk groups. Statistically significant DEGs were defined as those with an adjusted *p* value (adj. *p*) < 0.05 and a |fold change (FC)| ≥ 2, and were visualized in volcano plots. To determine gene function enrichment, the human c5.go.bp gene set was downloaded from the MSigDB database. Genes were pre-ranked based on differential expression (log_2_FC), and a GSEA analysis of the c5.go.bp gene set was conducted using the GSEA () function in the “clusterProfiler” R package.

### 2.7. Plasmid Construction

The *PEX5* sgRNA sequence (sg*PEX5*-1: 5′ ttcgtgcggcagattggcga 3′; sg*PEX5*-2: 5′ acgagccaagtcagctatag 3′) was subcloned into the lentiCRISPRv2 vector. The shRNA sequences to knockdown *ACOT4*, *CROT*, *HMGCL*, *PIPOX*, *GSTK1*, or *CAT*, as listed in Table S1, were subcloned into the pLKO.1 vector. The DNA encoding the C-terminal tripeptide, SKL, was cloned in frame with the DNA encoding the GFP tag at the C terminus to generate the GFP-PTS1 construct. Similarly, the DNA encoding the nonapeptide sequence, RLQSIKDHL, was cloned in frame with the GFP tag at the N terminus to generate the PTS2-GFP construct. The coding regions for *ACOX1*, *HMGCL*, *PIPOX*, *GSTK1*, or *CAT* were cloned into the pEGFP-C1 construct with an in-frame N-terminal GFP tag.

### 2.8. Cell Culture and PEX5 Knockout Cell Establishment

293T and U251 cells were purchased from American Type Culture Collection and cultured in Dulbecco’s modified Eagle’s medium (DMEM) (Biological Industries, Kibbutz Beit-Haemek, Israel 01-052-1A), supplemented with 10% fetal bovine serum (FBS) (Biological Industries, 04-001-1A), 100 units/mL penicillin, and 100 μg/mL streptomycin (Thermo Fisher Scientific, Waltham, MA, USA, 15070063). The cells were incubated in a humidified 5% CO_2_ incubator at 37 °C.

To establish *PEX5* knockout cells, the *PEX5* KO targeting vector was co-transfected with psPAX2 (Addgene, 12,260) and pMD2.G (Addgene, Watertown, MA, USA, 12,259) into 293T cells. The virus in the culture media was filtered through a 0.45 μm filter and used to infect U251 cells. Forty-eight hours after infection, cells were selected with puromycin (1 μg/mL). The surviving cell population was digested and single cells were plated into 96-well plates. The single-cell populations were then expanded and subjected to *PEX5* knockout detection by DNA sequencing and Western blot.

### 2.9. qPCR

Total RNA was isolated from cells using TRIzol reagent (Thermo Fisher Scientific, Waltham, MA, USA, 15596026), and reverse transcription was performed using a cDNA reverse transcription kit (Thermo Fisher Scientific, Waltham, MA, USA, 4374966). qPCR was carried out using primers listed in Table S2 to detect the gene expression. GAPDH was used as an internal control to calculate gene expression.

### 2.10. Western Blot

Cells were lysed in RIPA buffer (50 mM Tris-HCl pH 7.4, 150 mM NaCl, 1 mM EDTA, 1% Triton X-100, 0.5% sodium deoxycholate, 0.1% SDS) supplemented with protease inhibitor cocktails (Roche, Basel, Switzerland, 05892970001). Equal amounts of proteins were separated by SDS-PAGE and transferred to nitrocellulose membranes of 0.45 µm pore size. The membranes were then blocked with 5% skim milk, followed by incubation with α-Actin (Sigma, Tokyo, Japan, A1978; dilution: 1:10,000) or α-PEX5 (Cell Signaling Technology, Danvers, MA, USA, 83020S; dilution: 1:1000) antibodies overnight at 4 °C. Subsequently, the membranes were incubated with dye 800-conjugated secondary antibodies (LICOR, Lincoln, NE, USA, 926-32211 or 926-68070; dilution: 1:1000) and the fluorescence signals were captured using a Licor Odyssey-CLx machine.

### 2.11. Immunofluorescence

Cells grown on cover slides were transfected with GFP fusion constructs using a homemade polycation PEI reagent. After 24 h, the cells were fixed with 4% paraformaldehyde and permeabilized with 0.5% Triton X-100. They were then blocked with 1% BSA and incubated with the primary α-GFP antibody (Proteintech, Wuhan, China, 50430-2-A; dilution: 1:200) overnight at 4 °C. Following a washing step with PBS, the cells were incubated with fluorescent dye-conjugated secondary antibodies (Thermo Fisher Scientific, A11034; dilution: 1:200) and stained with 1μg/mL DAPI to label the nuclei. The subcellular localization of proteins was visualized using an Olympus FV3000 (Olympus, Tokio, Japan) laser scanning confocal microscope.

### 2.12. CCK-8 Assays

To assess cell proliferation, cells were plated into 96-well plates at a density of 1500 cells/well. At the indicated days, cell viability was measured using a Cell Counting Kit-8 (CCK-8) assay. The absorbance was measured at 450 nm using a microplate reader (Multiskan FC, Thermo Fisher Scientific).

### 2.13. Colony Formation Assay

Cells were seeded in 6-well plates (200 cells/well) and maintained in growth medium for 14 days. Cell colonies were stained with 0.1% crystal violet solution. The colonies were photographed and the colony number was calculated.

### 2.14. Cell Cycle

Cells were digested with 0.25% trypsin into single cells and washed with ice-cold PBS. The cells were fixed with 70% ice-cold ethanol for 2 h and then washed with PBS before being resuspended in PI staining buffer (Beyotime, Shanghai, China, C1052). After a 30 min incubation at room temperature in the dark, the cells were subjected to flow cytometry analysis using a BD flow cytometer. The cell cycle distribution was analyzed using FlowJo software (version 10.0.7r2).

### 2.15. Wound-Healing Assay

Cells were plated in 6-well plates and allowed to reach approximately 90% confluence. A sterile 10 µL pipette tip was used to create a scratch wound, and the cells were washed twice with PBS buffer. The cells were then incubated in growth media, and the wound area was imaged at the indicated times using an inverted fluorescence microscope. The wound-healing rate was calculated as follows: (wound width at 0 h—wound width at the indicated time)/wound width at 0 h.

### 2.16. Transwell

Cells were cultured in DMEM supplemented with 1% FBS in the upper chamber of a polycarbonate filter membrane with an 8.0 µm pore size (Corning-Costar, Cambridge, MA, USA). The lower chamber was filled with DMEM supplemented with 10% FBS. After 36 h of incubation, non-invading cells on the upper membranes were carefully removed using cotton swabs. The migrated cells on the lower membranes were fixed with 4% polyoxymethylene and stained with a 0.1% crystal violet solution. Five randomly selected fields for each transwell membrane were photographed, and the numbers of migrated cells were recorded.

### 2.17. Statistical Analysis

Experimental data were analyzed at least three times. Statistical analysis was performed using GraphPad Prism software (version 8.0.2) or R packages. Data are presented as the mean ± SD. The statistical tests, including Student’s t test, the Wilcox test, and a log-rank test, are indicated in the respective figure legends. A *p*-value of <0.05 was considered statistically significant, and the significance levels were denoted as follows: * *p* < 0.05; ** *p* < 0.01; and *** *p* < 0.001.

## 3. Results

### 3.1. PEX5 Deletion Inhibits Cell Growth, Migration, and Invasion of Glioma Cells

**Figure 1:**
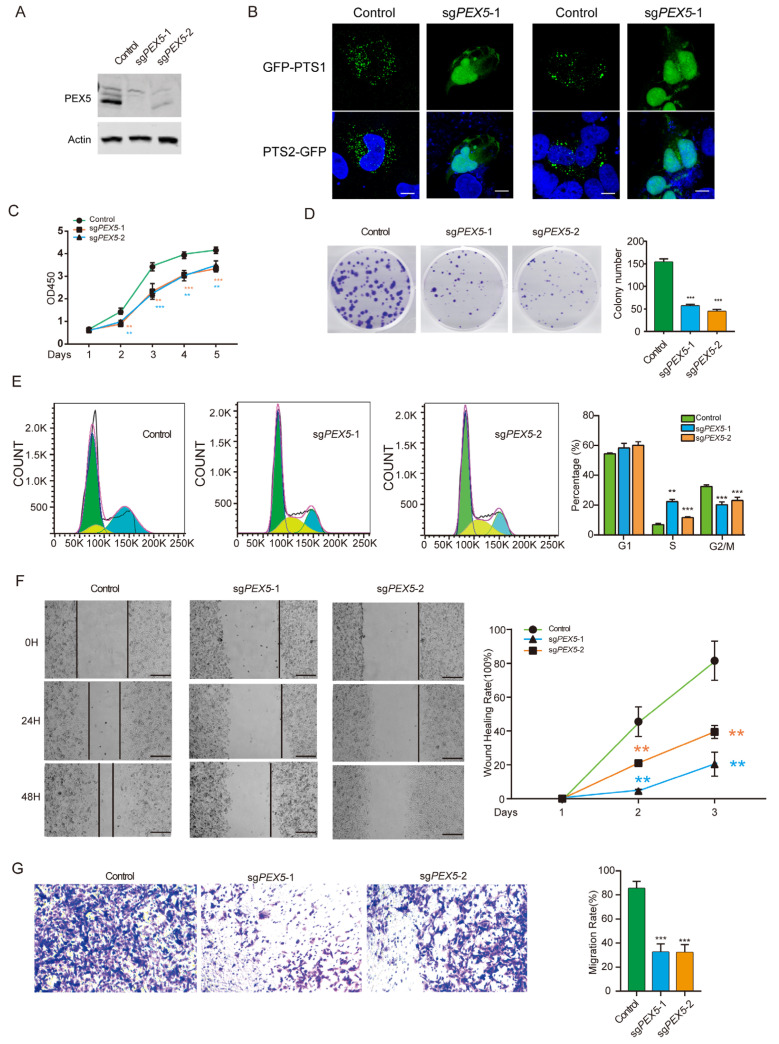
*PEX5* KO inhibits the proliferation, migration, and invasion of U251 cells. (**A**) Detection of *PEX5* in the control, sg*PEX5*-1, and sg*PEX5*-2 U251 cells (Original figures could be found in Supplementary File S1). (**B**) The control and sg*PEX5*-1 U251 cells were transfected with the GFP-PTS1 or GFP-PTS2 reporter constructs, or the indicated constructs. The cells were stained with GFP antibodies to enhance the fluorescent signal. Scale bars, 10 µm. U251 cells (control, sg*PEX5*-1, sg*PEX5*-2) were plated, and subjected to CCK8 (**C**), colony formation (**D**), flowcytometry (**E**), migration (**F**), and transwell (**G**) assays. Data are presented as the mean ± SD and representative of three independent experiments. ** *p* < 0.01; *** *p* < 0.001 (unpaired two-tailed Student’s *t* test). The yellow and blue asterisks in panel C indicated the *p* values for the sg*PEX5*-1 and sg*PEX5*-2 cells, respectively.

We designed two sgRNAs to target the *PEX5* gene and successfully knocked out (KO) *PEX5* in glioma U251 cells, as confirmed by the Western blot analysis (Figure 1A). PEX5 acts as a receptor for transporting PTS1-containing cargos into the peroxisomal matrix (9, 10), or as a co-receptor with PEX7 for the transport of PTS2 cargos (11, 12). Consistent with this role, we found that *PEX5* KO resulted in the defective import of PTS1- and PTS2-containing proteins, as demonstrated by the PTS1- and PTS2-reporter constructs (Figure 1B).

The CCK8 and colony formation assays revealed that *PEX5* KO severely suppressed the cell viability and proliferation (Figure 1C,D). Cell cycle analysis showed an increase and decrease in the percentage of cells in the S and G2/M phases, respectively, while the percentage of cells in the G1 phase was unchanged (Figure 1E). These findings suggest that *PEX5* KO inhibits cell growth by arresting the cell cycle from the S to the G2M phase. Furthermore, our wound-healing and trans-well assays demonstrated a remarkable impairment of migration and invasion in *PEX5* KO cells (Figure 1F,G). Overall, our results suggest that a deficiency in the import of peroxisomal matrix proteins due to *PEX5* knockout inhibits the cell growth, migration, and invasion of glioma cells.

### 3.2. Construction of A Prognostic Gene Signature in the TCGA Training Cohort

**Figure 2:**
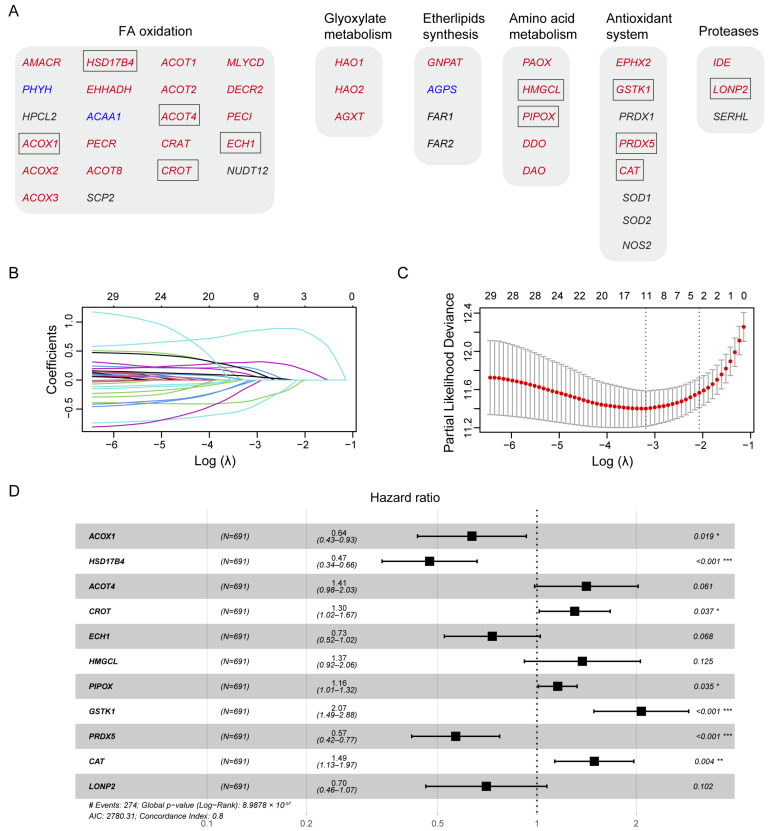
Identifying prognostic genes for developing a PEX5-dependent risk model. The six metabolic pathways in peroxisomes are depicted. The proteins with predicted PTS1 or PTS2 signals are denoted with red and blue fonts, respectively. The genes enclosed within the box represent the 11 signature genes selected by the LASSO model, as illustrated in panels (**B**,**C**) below. The 35 PTS1- or PTS2-containing genes in panel (**A**) were subjected to the LASSO coefficient analysis, using the TCGA dataset. The lines in different colors depict the coefficient distribution of each of the 35 genes. We selected the optimal parameter (lambda), as indicated by the first black dotted line, in the LASSO model in panel (**B**). (**D**) Forest plot of the 11 signature genes selected by the LASSO model. * *p* < 0.05; ** *p* < 0.01; *** *p* <0.001.

Human peroxisomal metabolic pathways play a critical role in various metabolic pathways, including fatty acid (FA) oxidation, glyoxylate metabolism, ether phospholipid biosynthesis, amino acid metabolism, antioxidant systems, and proteases. Using the peroxisome database (http://www.peroxisomeDB.org (accessed on 22 February 2024)), we identified 45 human peroxisomal enzymes involved in these pathways, 35 of which contained the predicted PTS1 or PTS2 signals (Figure 2A). Our findings demonstrate that PEX5-dependent metabolic pathways are significantly involved in glioma development (Figure 1). Therefore, we investigated whether the PTS1- or PTS2-containing metabolic enzymes, whose peroxisomal localization depends on PEX5, could be used to establish a prognostic model for glioma.

To develop a prognostic model for glioma, we used the TCGA glioma dataset as the training cohort and performed the least absolute shrinkage and selection operator (LASSO) Cox regression analysis on the 35 genes (Figure 2B,C). This resulted in a signature consisting of five genes (*ACOX1*, *HSD17B4*, *ACOT4*, *CROT*, *ECH1*), two genes (*HMGCL*, *PIPOX*), three genes (*GSTK1*, *PRDX5*, *CAT*), and the *LONP2* gene, which are involved in FA oxidation, amino acid metabolism, the antioxidant system, and proteases, respectively (Figure 2A,D).

Among these genes, *ACOX1*, *HSD17B4*, *ECH1*, *PRDX5*, and *LONP2* were identified as protective factors for glioma survival, as indicated by hazard ratios (HRs) smaller than 1 (Figure 2D). Conversely, *ACOT4*, *CROT*, *HMGCL*, *PIPOX*, *GSTK1*, and *CAT* were identified as risk factors, with HRs greater than 1 (Figure 2D).

### 3.3. Evaluation of the Prognosis Model

To assess the prognostic value of our PEX5-dependent model, we computed a risk score for each patient based on the mRNA expression levels of the 11 signature genes and LASSO Cox regression coefficients. Using the risk scores, we employed ROC curve analysis and Kaplan–Meier analysis to evaluate the sensitivity, specificity, and survival prediction of the model. In addition to the TCGA training cohort, we validated the model using two RNA-seq datasets (CGGA_693 and CGGA_325) and a microarray glioma dataset (GSE16011).

**Figure 3:**
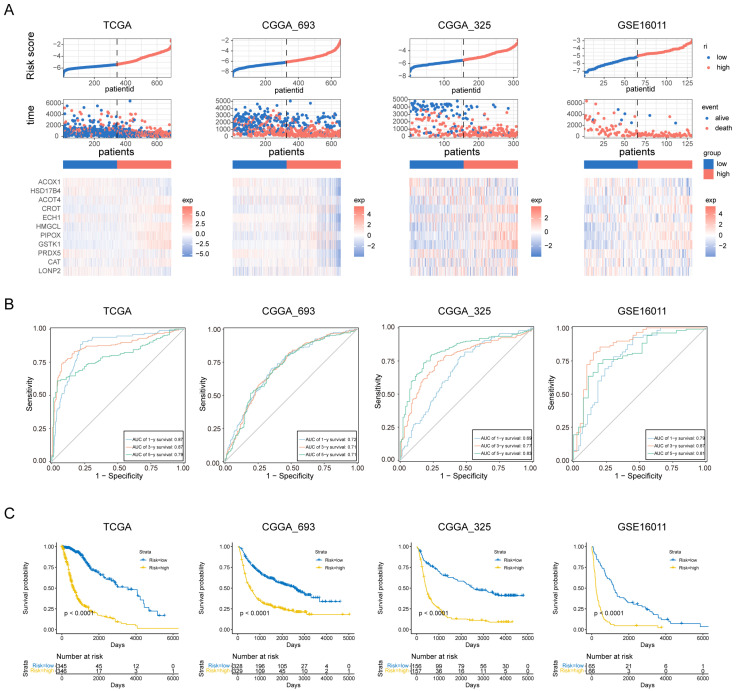
Assessment of the prognostic prediction ability of the 11-gene signature. (**A**) Distributions of risk scores, OS, and survival status, and heatmaps of gene expression profiles of the signature genes in the indicated cohorts. The dotted line indicates that patients were median-dichotomized into the low-risk group and high-risk group. (**B**) ROC curves of the 11-gene signature in the training TCGA dataset, and the three validation datasets: CGGA_693, CGGA_325, and GSE16011. (**C**) KM curve of the prognosis signature in the indicated four cohorts (log-rank test). The glioma patients were divided into the low- and high-risk groups based on the median signature score.

Based on the median risk score, we divided patients in each cohort into low- and high-risk groups. Figure 3A depicts the distribution of risk scores and survival times for each patient, along with the expression levels of the signature genes. Our analysis demonstrated that the PEX5-dependent model has excellent sensitivity and specificity for predicting 1-, 3-, and 5-year OS in all four cohorts, with area under curve (AUC) values of 0.87, 0.87, and 0.79 for the TCGA cohort, 0.72, 0.71, and 0.71 for the CGGA_693 cohort, 0.69, 0.77, and 0.83 for the CGGA_325 cohort, and 0.79, 0.87, and 0.81 for the GSE16011 cohort, respectively (Figure 3B). Furthermore, KM analysis showed that patients in the high-risk group had worse outcomes than those in the low-risk group across all cohorts (Figure 3C). These results suggest that our model has a strong predictive power.

Different clinicopathological features have been shown to influence the survival outcomes of glioma patients. For instance, patients with a higher World Health Organization (WHO) grade or *IDH* wild-type status are typically associated with a poor prognosis [24,25]. We examined the four cohorts containing the patient grade information and observed significantly higher risk scores among the GBM population (WHO grade IV) (Figure S1A). Additionally, the three RNA-seq cohorts included patient information on *IDH* and 1p19q codeletion status, and our analysis showed that patients with wild-type *IDH* or 1p19q non-codeletion status had significantly higher risk scores in these cohorts (Figure S1B,C). These findings highlight the prognostic value of the PEX5-dependent signature and demonstrate its ability to differentiate between different clinicopathological features in glioma patients.

### 3.4. Construction and Validation of the Nomogram

**Figure 4:**
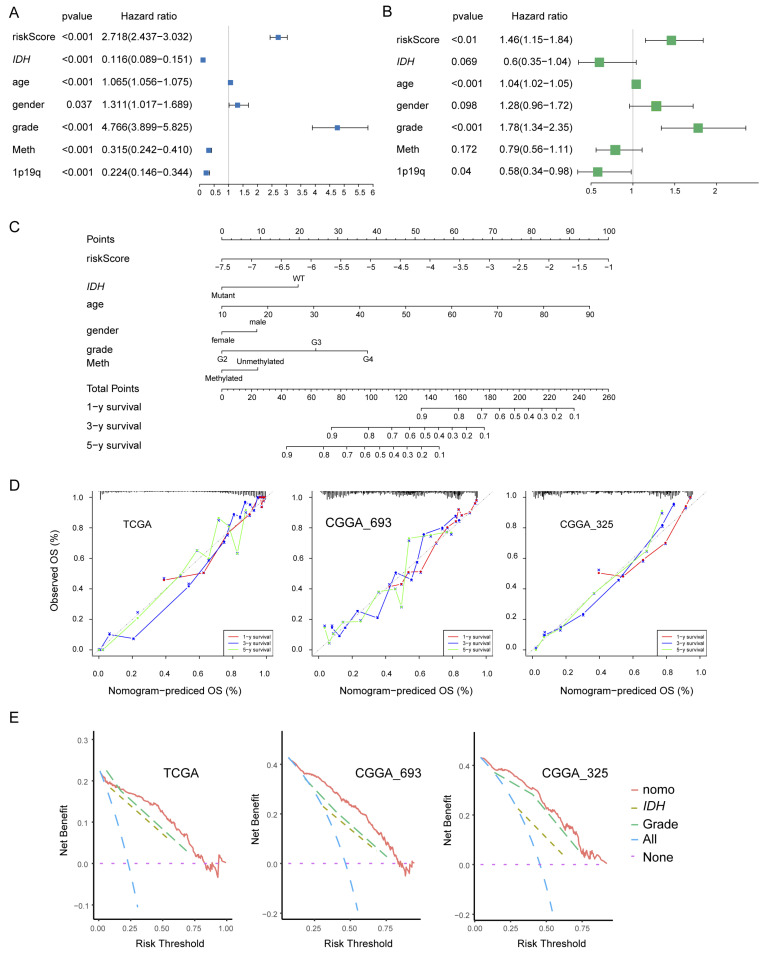
Establishment and assessment of the nomogram. (**A**,**B**) Forest plot of the univariate (**A**) and multivariate (**B**) Cox regression analyses of the indicated parameters in the TCGA cohort. *IDH*, *IDH* mutation status; grade, WHO grade; 1p19q, 1p19q codeletion status; Meth, MGMT promoter methylation status. The *X*-axis indicates the hazard ratio values. A hazard ratio of 1 signifies no difference in survival risk. (**C**) The nomogram plot was constructed based on the risk score, *IDH*, age, gender, grade, and Meth. The points of the factors indicate their corresponding contribution to the survival probability. The total points of each patient provide the estimated 3-year and 5-year survival times. (**D**) Calibration plot of the nomogram based on the TCGA, CGGA_693, and CGGA_325 datasets. (**E**) DCA of the nomogram for the 1-, 3-, and 5-year OS in the TCGA, CGGA_693, and CGGA_325 datasets.

We examined the independence of the PEX5-dependent risk score from other known prognostic factors, such as age, gender, WHO grade, *IDH* mutation status, 1p19q codeletion status, and MGMT promoter methylation status. Using univariate Cox analysis, we found that the PEX5-dependent risk score, along with other prognostic factors, was significantly associated with OS in the TCGA training cohort (Figure 4A). Multivariate Cox regression analysis revealed that the PEX5-dependent risk score, along with age, grade, and 1p19q codeletion status, were significantly correlated with OS (Figure 4B). These findings suggest that the risk score is an independent prognostic variable for glioma patients in the TCGA training cohort. To validate our findings, we extended our analysis to two independent cohorts (CGGA-325 and CGGA_693), for which clinicopathological parameters were available. Consistently, our results indicated that the PEX5-based risk score was an independent predictor of OS across these cohorts (Figure S2A,B,D,E).

We developed nomograms to predict 1-, 3-, and 5-year OS in glioma patients using the TCGA cohort. The nomograms incorporated seven independent risk factors, including the PEX5-dependent risk score, age, gender, WHO grade, *IDH* mutation, 1p19q codeletion, and MGMT promoter methylation statuses (Figure 4C). In the model, each risk factor was assigned points based on its contribution to OS (Figure 4C). Calibration curves indicated a good match between the actual and predicted OS at the 1-, 3-, and 5-year intervals (Figure 4D).

The clinical benefits of the nomogram were compared to those of the *IDH* mutation or WHO grade. DCA curves demonstrated that the nomogram exhibited superior predictive capabilities for OS compared to the *IDH* mutation or WHO grade. This superiority was observed across various threshold probabilities in both the training cohort (TCGA) and the validation cohorts (CGGA_693 and CGGA_325). Furthermore, the nomogram consistently yielded higher net benefits when compared to both the treat-all-patients (All) scheme and the treat-none (None) scheme (Figure 4E).

We constructed the nomogram model for the CGGA-693 and CGGA-325 cohorts by integrating six risk factors, including the risk score, age, gender, WHO grade, *IDH* mutation, and 1p19q codeletion statuses (Figure S2C,F). Since the majority of patients in these cohorts lacked information for the MGMT promoter methylation status, we omitted it from the nomogram construction. Consistently, these models demonstrated accurate prediction of patient survival for the 1-, 3-, and 5-year OS in both the CGGA-693 and CGGA-325 cohorts (Figure 4D,E).

### 3.5. High-Risk Group Had An Immunosuppressive Feature

**Figure 5:**
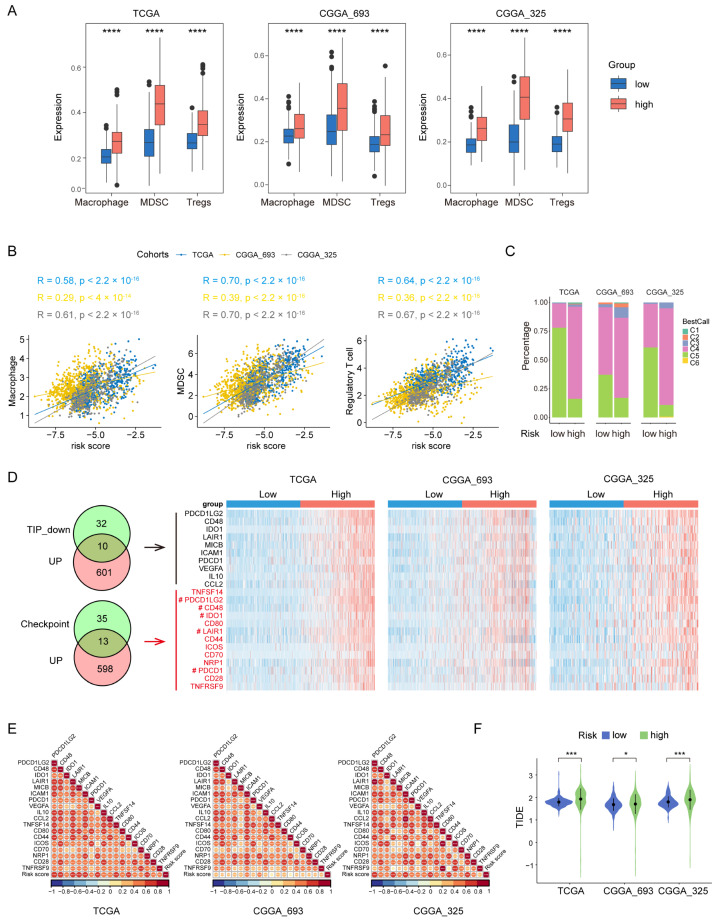
Comparisons of immunosuppressive features between the low- and high-risk gliomas. (**A**) The abundance of macrophages, MDSCs, and Tregs is compared between the low- and high-risk groups in the three cohorts. (**B**) The correlation of the risk score with the abundance of macrophages, MDSCs, and Tregs is demonstrated in the three cohorts. Pearson correlation was used to calculate the correlation coefficient (R) and p-values. (**C**) The distribution of immune subtypes is compared between the low- and high-risk groups in the three cohorts. (**D**) Intersection analysis of commonly upregulated genes in the high-risk group among the three cohorts with genes that inhibit the cancer-immunity cycle (TIP_down) or immune checkpoints. Heatmaps depict the expression levels of these overlapping genes between the low- and high-risk groups in the three cohorts. Genes that belong to both the cancer-immunity cycle inhibitors and checkpoint gene categories are denoted by the “#” label. (**E**) Correlation matrix visualization showing the relationship between the risk score and the overlapping genes from panel (**D**). Pearson correlation coefficient (R) values are indicated by the color scale. * *p* < 0.05; *** *p* < 0.001; **** *p* <0.0001. (**F**) Comparison of the TIDE scores between the low- and high-risk groups in the three cohorts. * *p* < 0.05; *** *p* < 0.001 (Wilcoxon test).

Gliomas exhibit a distinct tumor microenvironment, largely due to the presence of the blood–brain barrier [26]. During glioma progression, the infiltrating macrophages, Tregs and MDSCs have been shown to have protumor and immunosuppressive effects [26,27]. To examine the potential role of these immune cells in the prognosis value of the PEX5-dependent model, we computed the abundance of the three types of immune cells in each glioma patient through ssGSEA [28]. Our analysis revealed that the abundance of all three cell populations was significantly upregulated in the high-risk group and positively correlated with the risk score in the three examined cohorts (TCGA, CGGA_693, and CGGA_325) (Figure 5A,B).

We divided the glioma samples into different immune subtypes (C1–C6 subtypes) and found that the high-risk group had significantly more C4 and less C5 immune subtypes than the low-risk group in all three cohorts (Figure 5C). It is worth noting that the prognosis of the C4 immune subtype in tumors is worse than that of the C5 immune subtype [29]. These results suggest that the enhanced activity of the macrophages, Tregs, and MDSCs may contribute to the immunosuppressive microenvironment and worse prognosis in the high-risk group.

We analyzed the negative immune regulators, such as cancer-immunity cycle inhibitors [30] and immune checkpoints [31]. Through differential gene expression analysis through the limma package, we found that 567 and 611 genes were downregulated and upregulated, respectively, in the high-risk group across all three RNA-seq cohorts (Figure S3A,B). The Venn analysis showed that 10 cancer-immunity cycle inhibitors and 13 checkpoint genes were among the upregulated gene list (Figure 5D). Remarkably, five genes (*PDCD1LG2*, *CD48*, *IDO1*, *LAIR1*, and *PDCD1*) were found to be present in both the cancer-immunity cycle inhibitors and checkpoint genes categories. Overall, we identified 18 upregulated negative regulators (*PDCD1LG2*, *CD48*, *IDO1*, *LAIR1*, *MICB*, *ICAM1*, *PDCD1*, *VEGFA*, *IL10*, *CCL2*, *TNFSF14*, *CD80*, *CD44*, *ICOS*, *CD70*, *NRP1*, *CD28*, and *TNFRSF9*) in the high-risk group. Notably, the expression of each of these negative regulators was positively correlated with the risk score (Figure 5E).

To demonstrate the immunotherapy sensitivity differentiated by the model, we used the Tumor Immune Dysfunction and Exclusion (TIDE) score [32]. Our analysis showed that the high-risk group had higher TIDE scores than the low-risk group (Figure 5F), suggesting that the high-risk group is more likely to resist immunotherapy than the low-risk group.

### 3.6. The Peroxisomal Localization of the Signature Genes Depends on PEX5

**Figure 6:**
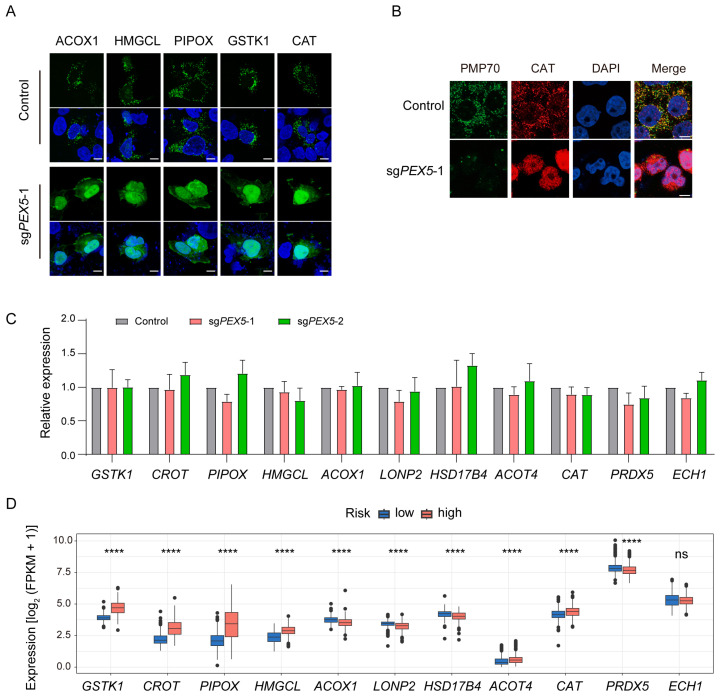
*PEX5* KO inhibits the peroxisomal localization of the signature genes. (**A**) The control and sg*PEX5*-1 U251 cells were transfected with the signature gene-expressing constructs. The cells were stained with GFP antibodies to enhance the fluorescent signal. Scale bars, 10 µm. (**B**) The control and sg*PEX5*-1 U251 cells were co-stained with PMP70 and CAT antibodies. Scale bars, 10 µm. (**C**) The expression of signature genes in the control and *PEX5* KO U251 cells detected by qPCR. Data are presented as the mean ± SD from three independent experiments. (**D**) Expression of the signature genes in the gliomas from low- and high-risk patients. **** *p* < 0.0001; ns, not significant.

All eleven signature proteins exhibit a peroxisomal targeting signal (PTS) at the COOH terminal, as corroborated by the Peroxisome Database (http://www.peroxisomeDB.org, (accessed on 22 February 2024)) (Table S7). Consistently, their peroxisomal localizations (ACOX1 [33], LONP2 [34], GSTK1 [35], CROT [36], PIPOX [37], HMGCL [38], HSD17B4 [39], ACOT4 [40], CAT [41], PRDX5 [42], and ECH1 [43]) have been referenced. We have demonstrated that the peroxisomal localization of PTS-containing proteins is dependent on PEX5 (Figure 1B). To further investigate the role of the import receptor PEX5 in determining the subcellular localization of signature genes, we transfected *PEX5* KO cells with constructs expressing ACOX1, HMGCL, PIPOX, GSTK1, or CAT. We observed their peroxisomal localizations. However, the peroxisomal localizations were found to be disrupted in *PEX5* KO cells (Figure 6A). Furthermore, we performed immunostaining with a CAT antibody and observed that the endogenous CAT proteins lost their typical peroxisomal localization in the absence of *PEX5* (Figure 6B). These findings collectively underscore the pivotal role of PEX5 in governing the peroxisomal localization of the signature genes.

We investigated the expression of the eleven signature genes using qPCR. Our results indicate that the expression levels of these genes remained unaffected in the *PEX5* knockout (KO) cells (Figure 6C). This underscores that our prognosis model is based on the reliance of the signature proteins on PEX5 for their peroxisomal localization, rather than their expression levels. Supporting this observation, we noted a distinct expression pattern in low- and high-risk glioma samples. In the high-risk group, *GSTK1*, *CROT*, *PIPOX*, *HMGCL*, *ACOT4*, and *CAT* exhibited elevated expression levels, while *ACOX1*, *LONP2*, *HSD17B4*, and *PRDX5* showed reduced expression levels (Figure 6D).

### 3.7. GSTK1 Inhibits the Growth, Migration, and Invasion of Glioma Cells

**Figure 7:**
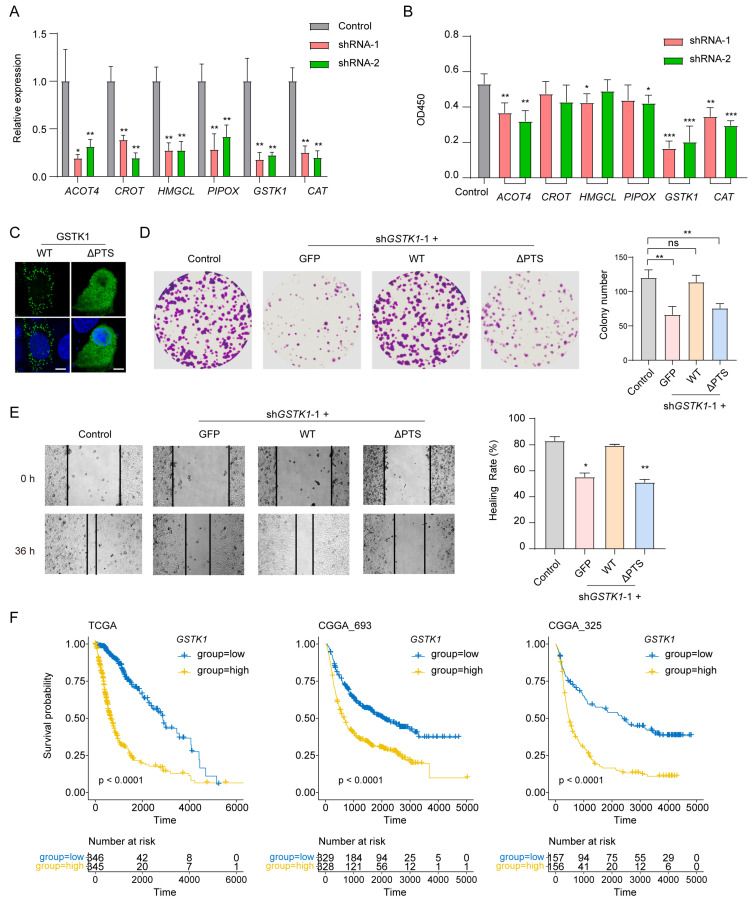
*GSTK1* silencing inhibits the proliferation and migration of U251 cells. (**A**) Evaluation of knockdown efficiency in U251 cells expressing the indicated shRNAs through qPCR. Two shRNAs were employed for each gene silencing. (**B**) Cell viability assay using CCK8 on cells from panel (**A**). (**C**) Subcellular localization of GFP-GSTK1 (WT) and GFP-(ΔPTS) proteins; scale bars, 10 µm. (**D**,**E**) The effect of *GSTK1* silencing on the colony formation and cell migration. (**F**) Glioma patients with a high expression of *GSTK1* had a reduced survival time. The glioma patients were divided into low- and high expression groups based on the median expression value of *GSTK1*. Data are presented as the mean ± SD and are representative of three independent experiments. * *p* < 0.05; ** *p* < 0.01; *** *p* < 0.001 (unpaired two-tailed Student’s *t* test).

Within our prognostic model, *ACOT4*, *CROT*, *HMGCL*, *PIPOX*, *GSTK1*, and *CAT* emerged as significant risk factors. To validate our prognostic model, we individually silenced these risk-factor genes in the glioma cell line U251 (Figure 7A). Our results demonstrate that the knockdown of *GSTK1*, an antioxidant enzyme, robustly inhibited the cell viability of glioma cells. The knockdown of *ACOT4* and *CAT* showed a comparatively less-pronounced reduction in cell viability, while the knockdown of other genes had marginal effects (Figure 7B). It is essential to emphasize that the identification of these risk factors relied on RNA-seq expression values obtained from bulk tumor samples, encompassing various cell types [44]. The distinct effects exhibited by these risk-factor genes may underscore their diverse functions within different cell types present in the glioma tumor. We therefore focused our further functional validation on *GSTK1* in U251 cells.

Subsequent experiments revealed that *GSTK1* silencing impairs the colony formation and cell migration of U251 cells (Figure 7D,E). To investigate whether these effects are dependent on peroxisomal localization, we re-expressed GSTK1 (wild type, WT) or a mutant with the deletion of the COOH-terminal peroxisomal targeting signal (ΔPTS) in *GSTK1*-knockdown cells. Our findings indicate that ΔPTS failed to localize in the peroxisomes (Figure 7C) and was unable to rescue the cell viability, colony formation, and cell migration as effectively as GSTK1 (WT) did (Figure 7D,E). Furthermore, the analysis of patient data from the TCGA, CGGA_693, and CGGA_325 glioma cohorts revealed that individuals with a high expression of *GSTK1* experienced reduced survival times (Figure 7F). These observations provide additional validation for the prognostic significance of our model and posit GSTK1 as a pivotal effector in the context of PEX5’s role in cell growth, migration, and invasion.

## 4. Discussion

Within our prognostic model, several signature genes are involved in FA oxidation (FAO) and redox metabolism, alterations of which have been observed during glioma progression and are associated with malignancy [14]. It is worth noting that these metabolic processes also occur in mitochondria, albeit with different enzymes and reactions involved [45]. Taking the FAO for example, two types of FAO occur in mammalian cells: α-oxidation and β-oxidation. α-oxidation is a unique process that takes place in peroxisomes and is responsible for removing a single carbon from certain lipids at the carboxy terminus, enabling their subsequent β-oxidation [46]. Very long-chain fatty acids (VLCFAs), which exceed 22 carbons in length, cannot be metabolized in the mitochondria and must undergo catabolism in peroxisomes [47]. While most of the research on FAO regards β-oxidation within the mitochondria in glioma cells [48,49], the study of peroxisomal FAO is very limited. Our PEX5-dependent prognosis model takes advantage of the unique peroxisomal metabolism and highlights the pivotal role of the PEX5-dependent peroxisomal metabolism in glioma development.

Some signature genes acted as protective factors while others posed risks for glioma survival. Notably, our results indicate that PEX5 promotes the cellular growth, migration, and invasion of glioma cells. The deletion of *PEX5* hampers the enzymatic activity of various peroxisomal proteins, suggesting that the observed effects might stem from the collective loss of function in both protective and risk-associated factors, or other PEX5-related targets. It is crucial to highlight that the identification of protective or risk factors was based on RNA-seq expression values derived from bulk tumor samples, encompassing not only cancer cells but also immune cells and other cell types [44]. By contrast, our functional analysis of *PEX5* knockout was conducted in the glioma cancer cell line U251. The distinct roles exhibited by PEX5 and these signature genes could highlight the varied functions of PEX5 targets within different cell types present in the glioma tumor.

Gliomas present a complex and unique immune microenvironment, largely due to the presence of the blood–brain barrier [26]. Within this microenvironment, various immune cell populations coexist, including macrophages, resident microglia, MDSCs, T cells, natural killer (NK) cells, and a small number of B cells [27]. Studies have shown that macrophages, Tregs, and MDSCs exert protumor and immunosuppressive effects during the progression of gliomas [26,27]. In gliomas, there is a noticeable shift in the polarization of tumor-associated macrophages (TAMs) from an M1 phenotype to an M2 phenotype, resulting in the suppression of the local immune response [50,51]. Recent research has linked TAMs to the promotion of tumor cell proliferation and the creation of an immunosuppressive environment through the recruitment of Tregs and MDSCs [52,53]. Furthermore, MDSCs contribute to the maintenance of an immunosuppressive environment by inhibiting various effector cells and promoting T-regulatory cell function [53].

Our findings indicate that patients in the high-risk group exhibit an immunosuppressive microenvironment characterized by the upregulation of macrophages, Tregs, and MDSCs, as well as the presence of certain negative immune regulators. Moreover, these patients demonstrate higher TIDE scores, indicating reduced sensitivity to immunotherapy. These findings offer insights into the potential mechanisms underlying the unfavorable prognosis associated with high-risk gliomas. Currently, immunotherapy has shown limited success in the treatment of glioma patients [27], and a major challenge lies in identifying the patients most likely to respond to this treatment. Therefore, our results provide valuable guidance for the development of personalized immunotherapy approaches for glioma patients.

Peroxisomes play a crucial role in compartmentalizing immune response pathways, serving as signaling platforms for initiating immune signaling cascades [54,55,56]. Additionally, peroxisomes act as immune metabolic hubs, influencing cellular metabolites such as ROS and unsaturated fatty acids. This functionality enables them to regulate immune cell development and activation, and modulate inflammatory pathways across different immune cells and tissues [57,58,59,60]. An impairment of macrophage activity has been observed in cells deficient in peroxisome biogenesis genes, such as *PEX5* [57,59], or the antioxidant enzyme *CAT* [57]. These findings, in conjunction with our results, suggest that metabolic alterations may contribute to the establishment of an immunosuppressive microenvironment in high-risk gliomas. However, further research is necessary to identify the specific metabolic enzymes and products involved in this process.

## Data Availability

Data are contained within the article and Supplementary Materials.

## Supplementary Materials

The following supporting information can be downloaded at: https://www.mdpi.com/article/10.3390/biom14030314/s1. Figure S1: Distribution of risk scores in gliomas with different clinicopathological features; Figure S2: Establishment of the nomogram using the CGGA_693 or CGGA_325 cohort; Figure S3: Differential expressed genes between the low- and high-risk groups; Tables S1–S4: the clinicopathological characteristics of glioma patients in the TCGA (Table S1), CGGA_693 (Table S2), CGGA_325 (Table S3), and GSE16011 (Table S4) cohorts; Table S5: the shRNA sequences used to knockdown the signature genes; Table S6: the primer sequences for the qPCR detection; Table S7: the predicted PTS sequence within the eleven signature proteins.
