# Supplementary material for: Identification and Validation of a PEX5-Dependent Signature for Prognostic Prediction in Glioma
Source: Biomolecules. 2024 Mar 6;14(3):314. doi: 10.3390/biom14030314 (PMC10967733; doi:10.3390/biom14030314)
Supplement: Supplementary file 1 [file biomolecules-14-00314-s001.zip › Supplemental Figures.pdf]

Supplementary Figure legends

Fig. S1 Establishment of the nomogram using the CGGA_693 or CGGA_325 cohort.
(A, B, D, E) Forest plot illustrating the results of univariate (A, D) and multivariate (B, E) Cox regression analyses for the indicated parameters using the CGGA_693 (A, B) or CGGA_325 (D, E) cohort.
(C, F) Construction of the nomogram plot using the CGGA_693 (C) or CGGA_325 (F) dataset, incorporating the indicated parameters. IDH, IDH mutation status; grade, WHO grade; 1p19q, 1p19q codeletion status.

Fig. S2 Differential expressed genes between the low and high-risk groups

(A) Volcano plots depicting the upregulated (UP) and downregulated (DOWN) genes in the low vs. high-risk groups across three datasets. The cutoffs for identifying differentially expressed genes (DEGs) are indicated by dashed lines: adjusted p-value (adj. p) < 0.05 and $|\text{fold change (FC)}| \geq 1$.
(B) Intersection analysis of the UP and DOWN genes identified in the three cohorts.

Supplementary tables

Table S1-S4

The clinicopathological characteristics of glioma patients in the TCGA (Table S1), CGGA_693 (Table S2), CGGA_325 (Table S3), and GSE16011 (Table S4) cohorts were indicated.
The events "1" and "0" denote the deceased and alive status, respectively. Time indicated the duration (days) for which the patients are monitored to assess their survival outcomes. Risk score for each patient was calculated based on the expression and coefficient of the genes within the PEX5 dependent signature, as described in methods and materials.

Table S5

The shRNA sequences used to knockdown the signature genes

Table S6

The primer sequences for the qPCR detection.

Table S7

The predicted PTS sequence within the eleven signature proteins.

Fig. S1

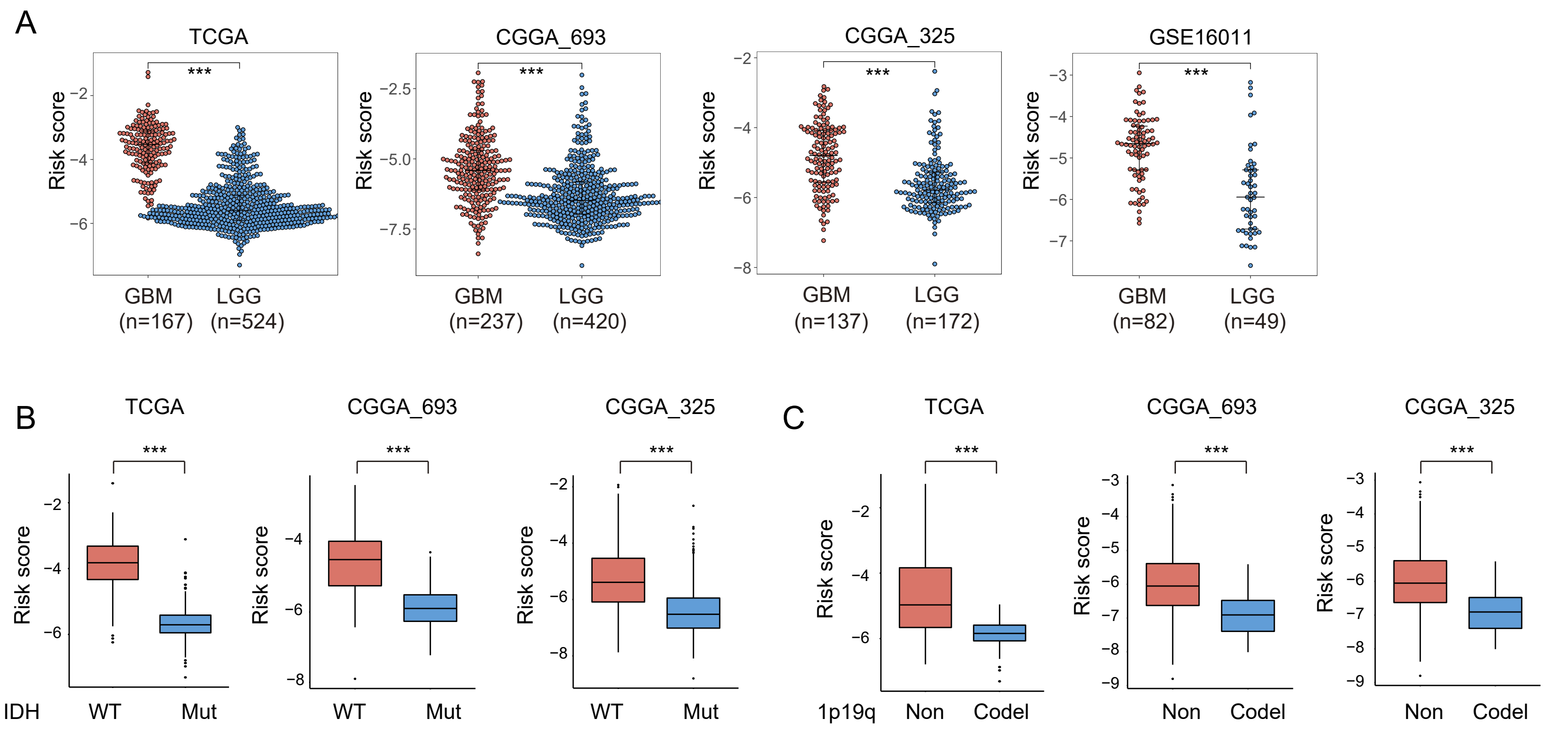
A
TCGA
CGGA_693
CGGA_325
GSE16011
Risk score
***
GBM
(n=167)
LGG
(n=524)
GBM
(n=237)
LGG
(n=420)
GBM
(n=137)
LGG
(n=172)
GBM
(n=82)
LGG
(n=49)
B
C
IDH
WT
Mut
1p19q
Non
Codel

Fig. S2

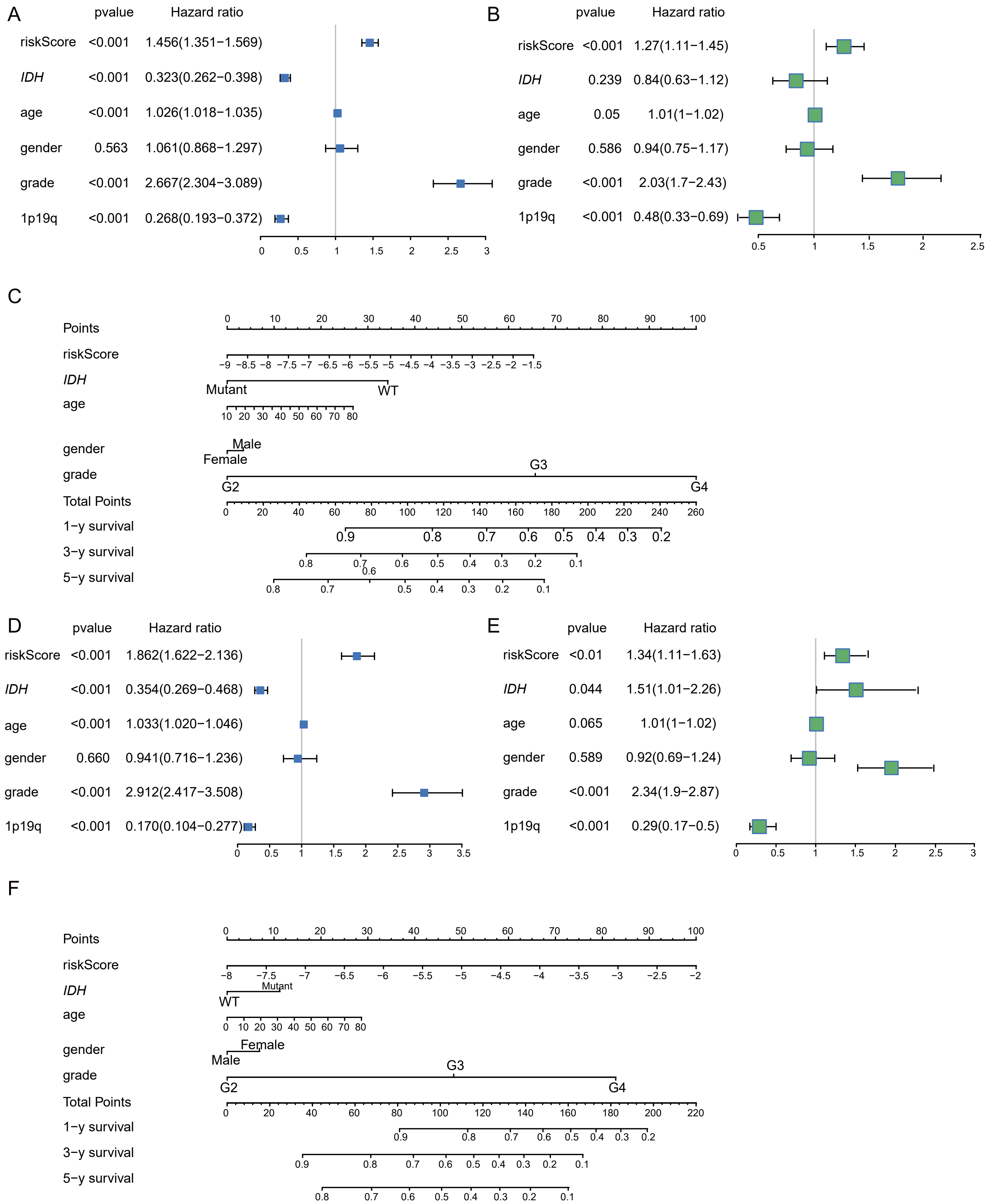
A
pvalue
Hazard ratio
riskScore <0.001 1.456(1.351−1.569)
IDH <0.001 0.323(0.262−0.398)
age <0.001 1.026(1.018−1.035)
gender 0.563 1.061(0.868−1.297)
grade <0.001 2.667(2.304−3.089)
1p19q <0.001 0.268(0.193−0.372)
B
pvalue
Hazard ratio
riskScore <0.001 1.27(1.11−1.45)
IDH 0.239 0.84(0.63−1.12)
age 0.05 1.01(1−1.02)
gender 0.586 0.94(0.75−1.17)
grade <0.001 2.03(1.7−2.43)
1p19q <0.001 0.48(0.33−0.69)
C
Points
riskScore
IDH
Mutant
WT
age
gender
Male
Female
grade
G2
G3
G4
Total Points
1−y survival
3−y survival
5−y survival
D
pvalue
Hazard ratio
riskScore <0.001 1.862(1.622−2.136)
IDH <0.001 0.354(0.269−0.468)
age <0.001 1.033(1.020−1.046)
gender 0.660 0.941(0.716−1.236)
grade <0.001 2.912(2.417−3.508)
1p19q <0.001 0.170(0.104−0.277)
E
pvalue
Hazard ratio
riskScore <0.01 1.34(1.11−1.63)
IDH 0.044 1.51(1.01−2.26)
age 0.065 1.01(1−1.02)
gender 0.589 0.92(0.69−1.24)
grade <0.001 2.34(1.9−2.87)
1p19q <0.001 0.29(0.17−0.5)
F
Points
riskScore
IDH
Mutant
WT
age
gender
Female
Male
grade
G2
G3
G4
Total Points
1−y survival
3−y survival
5−y survival

Fig. S3

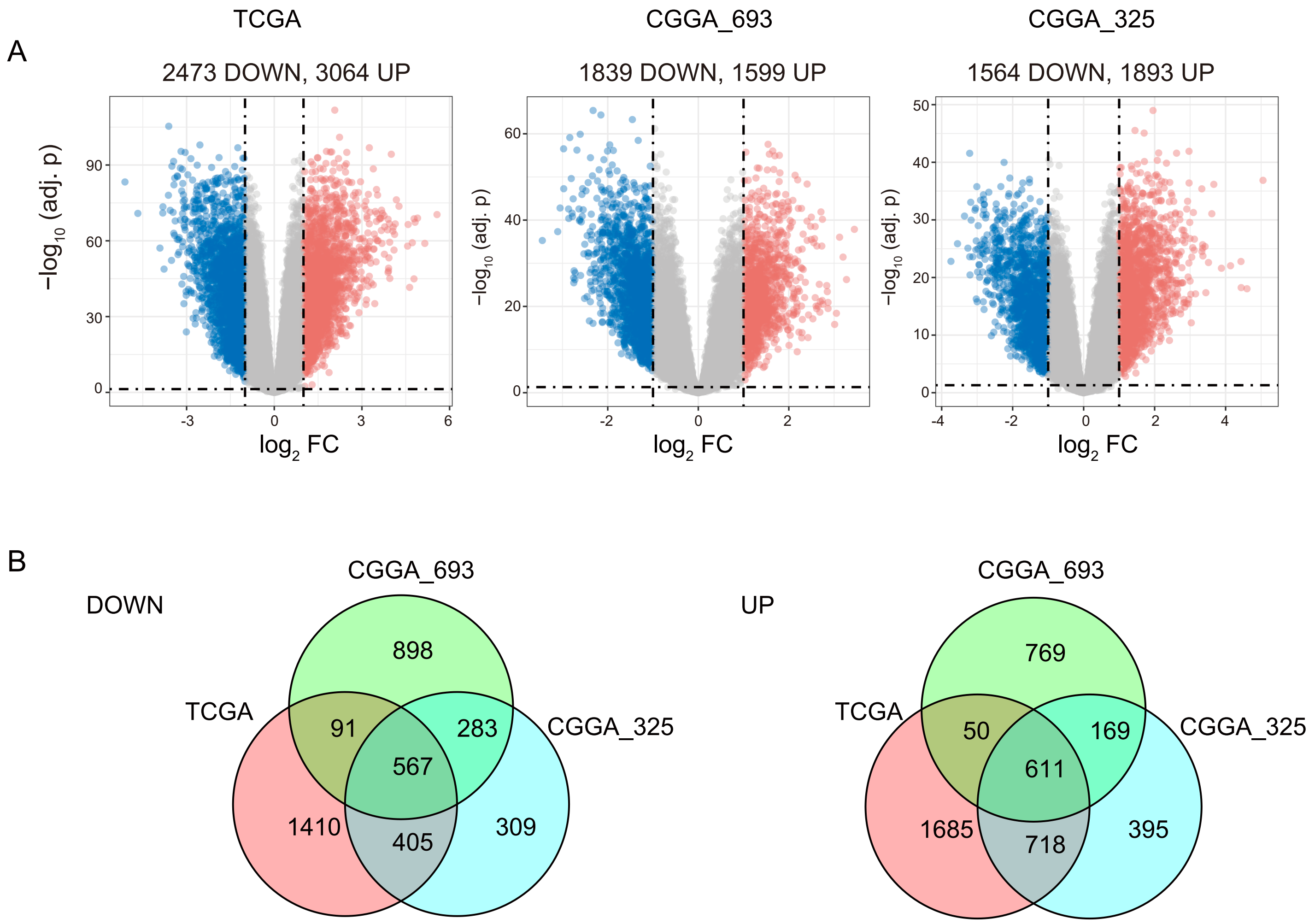
A
TCGA
2473 DOWN, 3064 UP
CGGA_693
1839 DOWN, 1599 UP
CGGA_325
1564 DOWN, 1893 UP
$-\log_{10}$ (adj. p)
$\log_2$ FC
B
DOWN
CGGA_693
TCGA
CGGA_325
898
91
567
283
1410
405
309
UP
CGGA_693
TCGA
CGGA_325
769
50
611
169
1685
718
395
