# Supplementary figures and images for: Identification and Validation of a PEX5-Dependent Signature for Prognostic Prediction in Glioma
Source: Biomolecules. 2024 Mar 6;14(3):314. doi: 10.3390/biom14030314 (PMC10967733; doi:10.3390/biom14030314)

Figure 1A

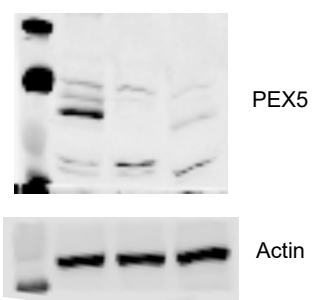

Supplement: Supplementary file 1 [file biomolecules-14-00314-s001.zip › supplementary file S1.pdf]
